# The complete chloroplast genome sequence of *Horsfieldia kingii* (Myristicaceae)

**DOI:** 10.1080/23802359.2019.1692722

**Published:** 2019-11-22

**Authors:** Xiao-Qin Li, Chang-Li Mao, Feng-Liang Zhang, Tian Yang, Qi Zhao, Yu Wu

**Affiliations:** Yunnan Institute of Tropical Crops, Xishuangbanna, China

**Keywords:** *Horsfieldia kingii*, chloroplast genome, Myristicaceae

## Abstract

*Horsfieldia kingii* is a member of Myristicaceae. The *H. kingii* chloroplast genome is found to be 155,655 bp in length and has a base composition of A (30.03%), G (19.52%), C (19.72%), and T (30.73%). The genome contained two short inverted repeat (IRa and IRb) regions (48,052 bp) which were separated by a large single copy (LSC) region (86,912 bp) and a small single copy (SSC) region (20,691 bp). The genome encodes 123 unique genes, including 85 protein-coding genes, 27 transfer RNA (tRNA) genes, and 8 ribosomal RNA (rRNA) genes. Further, complete chloroplast sequence of *H. kingii* was aligned together with other 2 species of Myristicaceae and other 5 basal angiosperms species which have reported the complete chloroplast sequence. This complete chloroplast genome will provide valuable information for the development of DNA markers for future species resource development and phylogenetic analysis of *H. kingii*.

Genus Horsfieldia, belonging to Myristicaceae, include about 5 genera (Editorial Committee of Chinese Academy of Sciences Flora 1979). For this genus, it is mainly studied on species classification (Wu et al. 2015), fatty acid ingredients (Xue and Fang 2012; Hu et al. 2010; Peng 2017), seed propagation (Hu et al. 2011), leaf traits variation (Hu et al. 2017), and rarely reported in molecular biology. *Horsfieldia kingii* is a species of genus Horsfieldia, it is mainly reported in chemical composition (Liu, Chen, et al. 2019; Liu, Du, et al. 2019), chlorophyII contents (Yang et al. 2018), but there is no reported in molecular biology else. In this study, we characterized the complete chloroplast genome sequence of *H. kingii* for phylogenetic analysis. The annotated genome sequence has been deposited Genbank under the accession number MK285562.

The fresh leaves of *H. kingii* was collected in 2017 from plantation base of Yunnan Institute of Tropical Crops (YITC), Jinghong, China (10°47′E, 22°00′N), and its genome DNA was extracted using the DNeasy Plant Mini Kit (QIAGEN, Valencia, CA), the remaining DNA was stored in an ultra-low temperature freezer. A specimen of this tree was conserved in YITC and the number of voucher specimen is 20140434. Genome sequencing was performed using Roche/454, sequencing libraries were prepared by the GS Titanium library preparation kit. The chloroplast genome assembled using CLC Genomic Workbench v3.6 (http://www.clcbio.com). The genes in the chloroplast genome were predicted using the DOGMA program (Wyman et al. 2004).

The circular genome is 155,655 bp in size, and comprises a large single copy (LSC) region (86,912 bp), a small single copy (SSC) region (20,691 bp), and two short inverted repeat (IRa and IRb) regions (48,052 bp). The base composition of the circular chloroplast genome is A (30.03%), G (19.52%), C (19.72%), and T (30.73%). The GC content of whole *H. kingii* chloroplast genome was 39.24%. The *H. kingii* chloroplast genome encodes a total of 123 unique genes, including 85 protein-coding genes, 27 transfer RNA genes, and 8 ribosomal RNA genes. There were 43 genes duplicated in the IR regions. The LSC region contained 64 genes, which including 43 protein-coding genes, 18 tRNA genes, and 2 rRNA genes, whereas 12 protein-coding genes and 2 tRNA gene were including in the SSC region. The introns were detected in 10 genes, *psbB*, *rpoB*, *rps7*, *atpH*, *ndhI*, *rpl23*, *rps19-fragment*, *trnQ-UUG*, *trnS-GGA*, *trnL-CAA* and they all have 1 intron.

To study *H. kingii* phylogenetic relationship with the angiosperms, *Horsfieldia pandurifolia, Myristica yunnanensis* of Myristicaceae (Changli, Fenglian, Tian et al. 2019; Changli, Fenglian, Xiaoqin et al. 2019) and other complete chloroplast genome sequences of angiosperms were download for analyses. The maximum likelihood phylogenetic was performed using MEGA X (Kumar et al. 2018) (Figure 1). A bootstrap analysis was performed on the resulting phylogenetic tree, and values were obtained after 1000 replications. The result shows that *H. kingii* was clustered with other species and closely to *Horsfieldia pandurifolia* and *Myristica yunnanensis* chloroplast complete genome.

**Figure 1.**
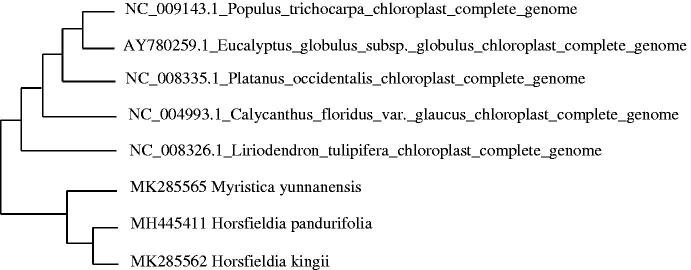
Maximum-likelihood phylogenetic tree of *H. kingii* with 7 species based on complete chloroplast genome sequences. The gene’s accession number is list in figure and the data of *H. pandurifolia* and *M. yunnanensis* come from author.
